# Alterations in DRBD3 Ribonucleoprotein Complexes in Response to Stress in *Trypanosoma brucei*

**DOI:** 10.1371/journal.pone.0048870

**Published:** 2012-11-08

**Authors:** Sandra M. Fernández-Moya, Angélica García-Pérez, Susanne Kramer, Mark Carrington, Antonio M. Estévez

**Affiliations:** 1 Instituto de Parasitología y Biomedicina “López-Neyra”, IPBLN-CSIC, Armilla, Granada, Spain; 2 Department of Biochemistry, University of Cambridge, Cambridge, United Kingdom; University of Texas-Houston Medical School, United States of America

## Abstract

Regulation of RNA polymerase II transcription initiation is apparently absent in trypanosomes. Instead, these eukaryotes control gene expression mainly at the post-transcriptional level. Regulation is exerted through the action of numerous RNA-binding proteins that modulate mRNA processing, turnover, translation and localization. In this work we show that the RNA-binding protein DRBD3 resides in the cytoplasm, but localizes to the nucleus upon oxidative challenge and to stress granules under starvation conditions. DRBD3 associates with other proteins to form a complex, the composition of which is altered by cellular stress. Interestingly, target mRNAs remain bound to DRBD3 under stress conditions. Our results suggest that DRBD3 transports regulated mRNAs within the cell in the form of ribonucleoprotein complexes that are remodeled in response to environmental cues.

## Introduction

Kinetoplastids, such as *Trypanosoma brucei*, *Trypanosoma cruzi* and *Leishmania* sp., are protozoa representing one of the deepest branches in the eukaryotic lineage [1]. Many of them are parasites, and have a significant impact on human health [2]. They exhibit many biological processes that are divergent from crown group eukaryotes, especially in gene expression [3]. Remarkably, genes are constitutively transcribed from long polycistronic units [4], and individual mRNAs are generated by coupled *trans*-splicing and polyadenylation reactions [5]. RNA polymerase II transcription initiation seems to be controlled by histone marks rather than by regulatory transcription factors [6]. Consequently, kinetoplastid gene expression is regulated primarily at the post-transcriptional level [3], [7], [8]. Trypanosomes and Leishmanias alternate between insect and mammalian hosts during their life cycles. Despite the absence of transcriptional control, they have to remodel gene expression to adapt to the drastic changes in pH, temperature, osmolarity, defences, nutrients and oxygen availability present within one or other host, or to different compartments within the same host [9], [10]. This is thought to be achieved through the concerted action of RNA-binding proteins (RBPs) that regulate mRNA maturation, translation, localization and degradation in response to environmental cues [3], [7], [11]. Although there are large numbers of predicted RBPs encoded in the genomes of kinetoplastids, only few have been functionally characterized [11], and virtually nothing is known about how RBPs are able to modulate the abundance of mRNAs and proteins. As reported in other eukaryotes [12], changes in the subcellular localization of several RBPs have been observed when trypanosomes are challenged *in vitro* to different stresses, such as heat shock, oxidative stress or starvation. *T. cruzi* uridine binding protein 1 (UBP1) is a destabilizing factor that binds to a specific group of mRNAs, and is found in a ribonucleoprotein complex associated with another RBP, UBP2 [13], [14]. Both UBP1 and UBP2 shuttle from the cytoplasm to the nucleus when the oxidative stress pathway is activated by sodium arsenite [15]. SR62, an RBP of the SR-related protein family, and the polypyrimidine tract binding protein PTB2, relocalize from nuclear speckles to the nucleolus upon heat shock in *T. cruzi* [16]. On the other hand, heat shock and starvation promote the accumulation of UBP1, UBP2, the poly(A)-binding proteins PABP1 and PABP2, the RNA-helicase DHH1, the translational repressor SCD6 and several other RBPs in cytoplasmic granules in *T. cruzi* and *T. brucei* [17], [18]. Importantly, UBP1 was also observed in cytoplasmic granules of trypanosomes isolated from the insect vector where nutrients may well be limiting [18]. However, we still have a very limited knowledge of how RBPs interact with target mRNAs and with other protein partners in response to environmental cues. The RNA-binding protein DRBD3 is one of the few characterized RBP in trypanosomes. This essential protein associates to a specific subset of mRNAs, promoting their stabilization in *T. brucei* [19], [20]. In this work we have analyzed the behavior of DRBD3 under different stress conditions. We show that DRBD3 is part of a multiprotein complex that undergoes changes in both the subcellular localization and composition upon arsenite or starvation-induced stresses.

## Results

### Arsenite and Starvation Stresses Cause Relocalization of DRBD3

**Figure 1:**
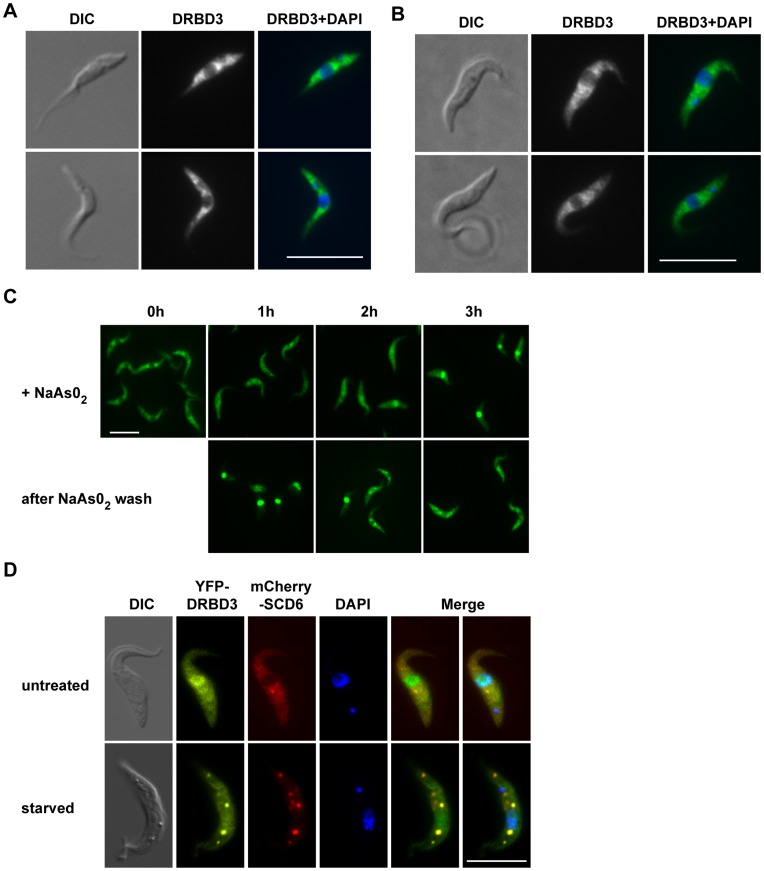
Effect of arsenite- and starvation-induced stress on DRBD3 localization. Immunofluorescence analyses were performed with anti-DRBD3 antiserum in wild-type cells (A) or with anti-protein A in trypanosomes expressing DRBD3 fused to the TAP tag (B). (C) Arsenite promotes the accumulation of DRBD3 in the nucleus. Cells were incubated in SDM-79 medium supplemented with 50 µM sodium arsenite for up to 3 hours. Aliquots were taken every hour and treated for immunofluorescence assays using anti-DRBD3 antiserum. Parasites were then washed in SDM-79 and incubated for 3 additional hours. (D) DRBD3 localizes to stress granules in starved trypanosomes. Cells co-expressing YFP-DRBD3 and mCherry-SCD6 were washed twice in phosphate-buffered saline and incubated in the same buffer for three hours. Bars, 10 µm.

We have previously shown that DRBD3 localization is mainly cytoplasmic using a specific antiserum in immunofluorescence analysis [19]. However, DRBD3 is not homogeneously distributed in the cytoplasm and accumulates in the perinuclear region (Figure 1A). We expressed a TAP tagged version of DRBD3 from the endogenous locus using a cell line, TAP-DRBD3/−, with the second allele replaced with an antibiotic resistance marker (Figure S1). TAP-DRBD3/− cells exhibited a generation time prolonged by about 20% with respect to wild-type trypanosomes, and showed an identical DRBD3 localization pattern (Figure 1B). We next analyzed whether DRBD3 distribution was altered by oxidative or nutritional stresses. As shown in Figure 1C, DRBD3 translocated to the nucleus when parasites were incubated in the presence of sodium arsenite. This phenomenon was reversible, since cells remained viable and DRBD3 returned to the cytoplasm when arsenite was removed from the culture medium. Most, but not all DRBD3 protein was detected in the nucleus after a 3 hours treatment with 50 µM arsenite. Longer incubation times caused the appearance of rounded cells and did not increase the nuclear accumulation of DRBD3 (Figure S2). DRBD3 relocalization was not simply due to a general shuttling of cellular RNA-binding proteins to the nucleus upon oxidative stress, since neither poly-A binding proteins PABP1/PABP2 nor the RNA helicase DHH1 accumulated in the nucleus even after 4 hours exposure to arsenite (Figure S2).

Nutritional stress, on the other hand, induced the accumulation of DRBD3 in cytosolic granules (Fig. 1D). Thus, upon incubation of trypanosomes in phosphate-buffered saline for 3 hours, cells remained viable and DRBD3 concentrated in discrete foci that colocalized with the stress granules marker SCD6 [17], [18]. These results indicate that DRBD3 localization within the cell can be modulated in response to different environmental stimuli.

### DRBD3 Associates with other Proteins to Form a Multiprotein Complex

**Figure 2:**
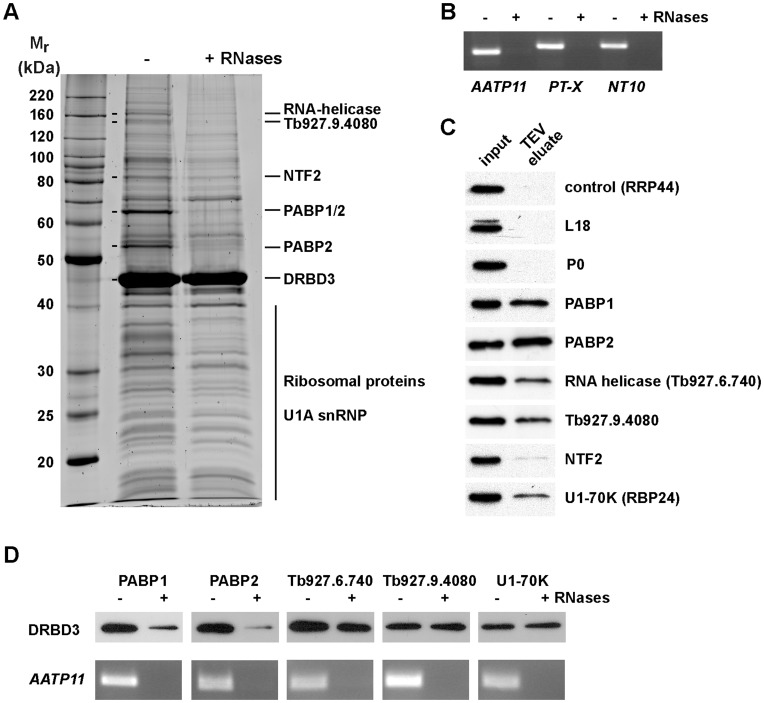
Identification of DRBD3-associated proteins by tandem-affinity purification (TAP). (A) Protein extracts were obtained from cells expressing a TAP-DRBD3 fusion from the endogenous locus. The lysate was split into two halves, and RNAses A and T1 were added to one. Both fractions were processed in parallel for TAP as described in Materials and Methods. (B) The efficiency of the RNAses treatment was assessed by RT-PCR of the purified material after TEV protease incubation using oligonucleotides specific for transcripts known to be bound to DRBD3. (C) Protein interactions were confirmed by generating cell lines expressing TAP- or PTP-tagged versions of some of the identified protein partners. The presence of DRBD3 was assayed in the purified material by western blot analysis after IgG chromatography and TEV protease incubation. Ribosomes were pulled down using either L18 or P0 ribosomal proteins as baits. (D) To assess whether the observed protein interactions were RNA-dependent, lysates obtained from cell lines expressing TAP- or PTP-tagged versions of the indicated proteins were treated with RNAses A and T1 as indicated above. The presence of DRBD3 was assayed in the TEV eluates as in panel C, and the efficiency of the RNases treatment was evaluated by RT-PCR using oligonucleotides specific for the DRBD3-bound transcript *AATP11* as above.

We used the tandem-affinity purification (TAP) method [21] coupled to mass spectrometry to analyze whether DRBD3 binds to other proteins in the cell. DRBD3 was found in a complex that included poly(A)-biding proteins PABP1 and PABP2, a putative RNA-helicase Tb927.6.740, a protein with a Zn finger domain (Tb927.9.4080), two U1 small nuclear proteins (U1-70K and U1C), a putative nuclear transport factor (Tb927.10.2240) and at least 20 ribosomal proteins from both large and small subunits (Figure 2A and Table 1).

**Table 1:** Proteins associated with DRBD3.

Systematic name	Description of gene product	kDa	Score
Tb927.10.1510	NOT1	259.1	74
**Tb927.9.4080**	Protein with Zn finger domain	142.1	146
**Tb927.6.740**	RNA helicase	139.1	150
**Tb927.6.3750**	HSP70	71.5	108
Tb927.10.2240	Protein with NTF2 domain	66.4	76
**Tb927.9.9290**	PABP1	63.0	99
**Tb927.9.10770**	PABP2	62.1	107
Tb927.4.1790	Ribosomal protein L3	54.4	58
Tb927.1.2340	Alpha-tubulin	49.8	91
**Tb927.1.2330**	Beta-tubulin	49.7	88
**Tb927.10.2100**	TEF1	49.1	50
**Tb927.3.5050**	Ribosomal protein L4	41.9	61
**Tb927.9.8740**	DRBD3	37.0	129
**Tb927.11.2060**	Ribosomal protein P0	34.6	54
**Tb927.8.4830**	RBP24 U170K	31.7	95
**Tb927.8.1330**	Ribosomal protein L7a	30.9	66
**Tb927.11.3590**	Ribosomal protein S4	30.6	131
**Tb927.9.6070**	Ribosomal protein S3	30.4	36
**Tb927.10.3940**	Ribosomal protein S3a	29.4	105
**Tb927.5.1110**	Ribosomal protein L2	28.3	88
**Tb927.7.1730**	Ribosomal protein L7	27.7	118
**Tb927.11.10790**	Ribosomal protein SA	27.6	71
Tb927.3.3320	Ribosomal protein L13	25.4	55
Tb927.9.8420	Ribosomal protein L10	24.7	84
**Tb927.7.5180**	Ribosomal protein L23a	24.7	66
**Tb927.10.13500**	Ribosomal protein L10a	24.6	37
Tb927.9.3990	Ribosomal protein S7	23.8	45
Tb927.10.12330	ZC3H34	22.4	57
**Tb927.9.7590**	Ribosomal protein L11	22.4	59
**Tb927.8.1110**	Ribosomal protein S9	22.1	36
**Tb927.10.2120**	U1A small nuclearribonucleoprotein	21.8	55
Tb927.6.720	Ribosomal protein L14	21.4	35
**Tb927.10.560**	Ribosomal protein S11	20.1	44
**Tb927.10.5370**	Ribosomal protein S10	19.3	20
Tb927.4.1860	Ribosomal protein S19	18.8	60

Proteins co-purifying with TAP-DRBD3 were excised from SDS-PAGE gels, subjected to MALDI-TOF analysis and identified using MASCOT software. Proteins indicated in boldface were detected at least in two independent purifications. MASCOT probability based Mowse scores are reported. Scores >51 are statistically significant (p<0.05).

**Figure 3:**
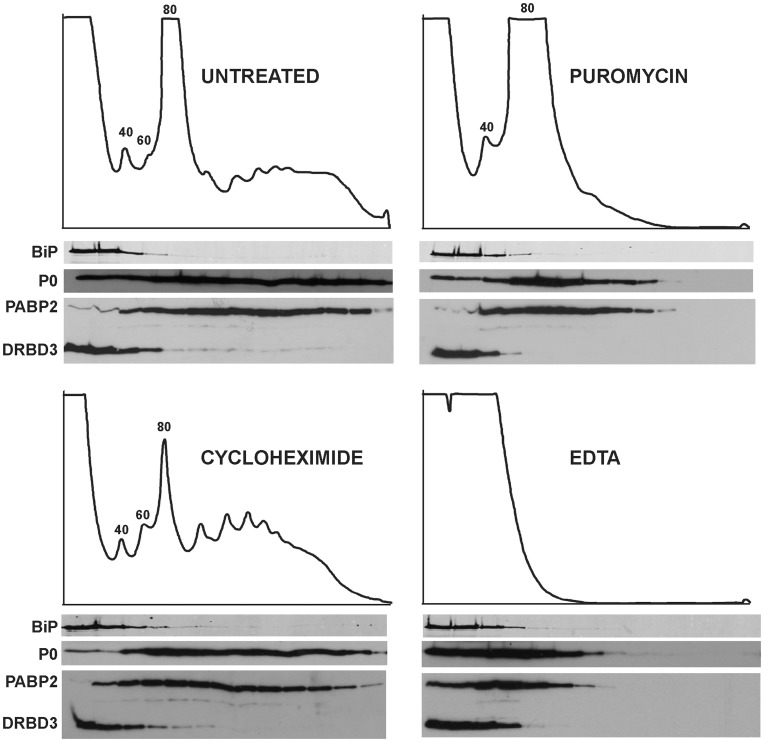
DRBD3 does not associate with polysomes. Trypanosome lysates were subjected to sucrose gradient separation in the absence or presence of translational inhibitors. Absorption at 254 nm was recorded during fractionation. Fractions were analyzed by western blot using an anti-DRBD3 antiserum. The distribution of DRBD3 along the gradients was compared to those of proteins known to be polysome-associated (ribosomal protein P0 and PABP2) or not bound to polysomes (BiP).

PABP1 and PABP2 comigrated in a single band with a mobility of 65 kDa. We always detected a second PABP2 polypeptide of ∼52 kDa that probably arises after proteolysis (Figure 2A, see also Figure S3B). Binding to both PABPs was disrupted when the protein extract was incubated with RNAse A and RNAse T1 prior to TAP (Figure 2A), indicating that the interaction of DRBD3 with PABP1 and PABP2 is RNA-dependent. To confirm the association of DRBD3 with some of the identified protein partners, we generated cell lines that expressed candidate proteins fused to the TAP- or the PTP [22] tags. The presence of bound DRBD3 was tested by western assays after IgG-sepharose chromatography and TEV protease cleavage. A cell line expressing a TAP-tagged version of the exonuclease RRP44 [23] was used as a negative control. We confirmed the interactions with PABP1, PABP2, the RNA helicase Tb927.6.740, the Zn finger domain protein Tb927.9.4080 and the U1A snRNP U1-70K (Figure 2C). Interaction with both PABPs was lost after treatment with RNases, as expected, whereas the association to other proteins in the complex was found to be RNase-resistant (Figure 2D). The presence of many ribosomal proteins in the TAP purified fractions suggested association to ribosomes. However, we were unable to detect DRBD3 in either TAP-purified ribosomes or in polysomes (Figure 2C and Figure 3).

### The Composition of DRBD3 Protein Complexes is Affected by Stress

**Figure 4:**
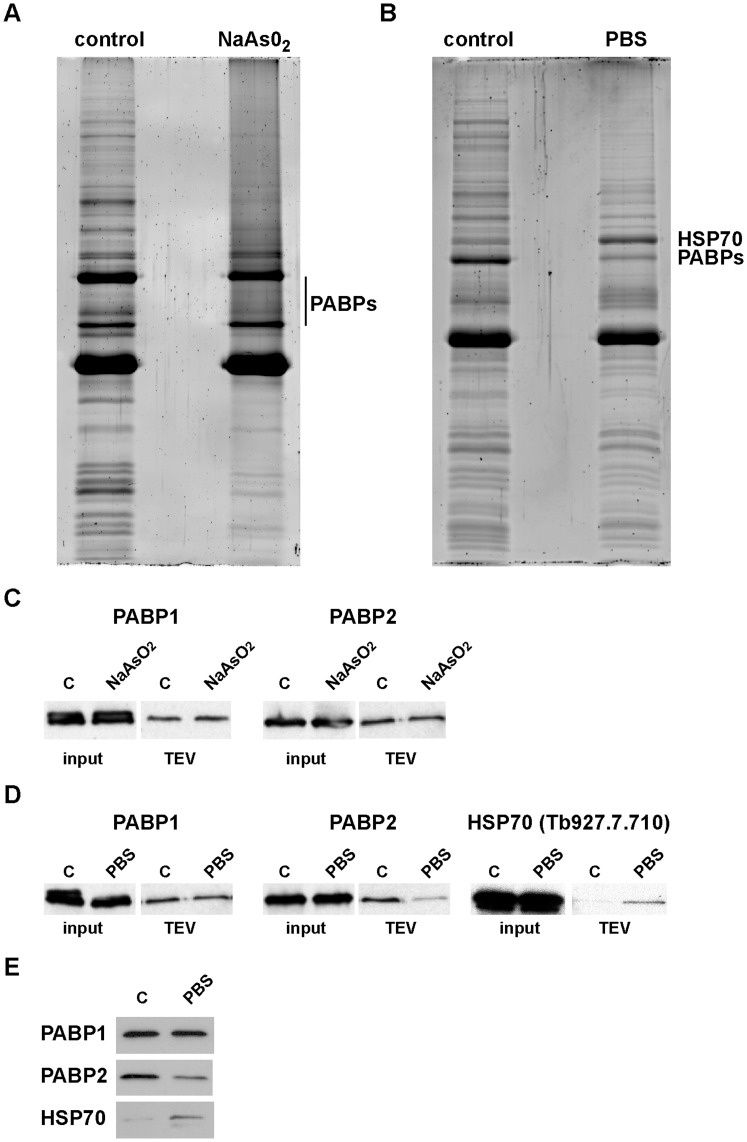
Arsenite- and starvation-induced stress affects DRBD3 complex composition. DRBD3 protein complexes were TAP-purified from cells incubated with 50 µM sodium arsenite for 3 h (A) or starved in PBS for 3 h (B), and compared to TAPs carried out with lysates obtained from equivalent amounts of normally grown trypanosomes (control). (C) Cell lines were generated that expressed both TAP-DRBD3 and 4xTy-tagged versions of PABP1 or PABP2. The presence of PABPs was assayed in the presence or absence of arsenite by western blot after IgG chromatography and TEV cleavage. (D) Association of Ty-PABP1, Ty-PABP2 and Ty-HSP70 with DRBD3 in starvation conditions was also analyzed as in panel C. (E) We also generated cell lines expressing TAP- or PTP-tagged versions of PABP1, PABP2 and HSP70. The amount of DRBD3 that copurified with these proteins in starvation conditions was analyzed after IgG chromatography and TEV cleavage using a DRBD3 antiserum.

Since cellular stress influences DRBD3 localization, we sought to determine whether the interaction of DRBD3 with its protein partners was also affected by starvation and oxidative challenges. DRBD3 protein levels remained constant when trypanosomes were treated with sodium arsenite or starved in phosphate-buffered saline (Figure 4, and data not shown). Upon arsenite treatment, PABP1 and PABP2 remained associated with DRBD3 to approximately the same degree as in control cells. The amount of most of the other co-purifying proteins was reduced (Figure 4A). To confirm these observations, trypanosomes expressing TAP-DRBD3 were modified to express Ty- epitope tagged versions of PABP1 and PABP2 from their endogenous loci, and the monoclonal antibody BB2 [24] was used to visualize these proteins by western blot analysis. As shown in Figure 4C, the amount of PABP1 and PABP2 associated with DRBD3 was similar in the presence or absence of arsenite. In contrast, on starvation stress the amount of PABP2, but not PABP1, that co-purified with DRBD3 was reduced, and the amount of one HSP70 (Tb927.7.710) that co-purified was increased (Figure 4B). This phenomenon was observed in several independent purifications, and was confirmed using Ty-tagged versions of both PABPs and HSP70 as indicated above (Figure 4D). The observed changes in the amount of PABP2 and HSP70 associated with DRBD3 were not due to variations in the total levels of these proteins, since they were similar before and after treatment (input). To further confirm these results, we used cell lines expressing TAP- or PTP-tagged versions of PABP1, PABP2 or HSP70, and analyzed the amount of DRBD3 bound to these proteins upon nutritional stress. As expected, the amount of DRBD3 that co-purified with PABP2 was reduced in starved trypanosomes, whereas the amount of DRBD3 associated with HSP70 was increased (Figure 4E). Although SCD6 colocalized with DRBD3 in stress granules (Figure 1), we could not detect it in the TAP purified material (data not shown).

### DRBD3 Remains Bound to Target mRNAs in Stress Conditions

**Figure 5:**
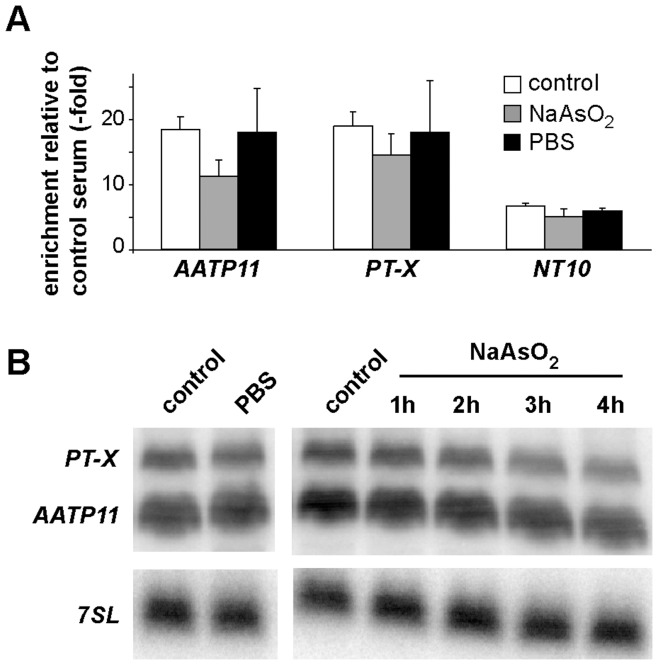
DRBD3-associated transcripts remain bound in stress conditions. (A) Association of DRBD3-regulated mRNAs was assayed by quantitative RT-PCR after immunoprecipitation of DRBD3 ribonucleoprotein complexes using anti-DRBD3 antiserum from normally grown (control) or stressed trypanosomes. Fold-enrichment of target transcripts was calculated after comparing to mock immunoprecipitations carried out using normal rabbit serum and normalizing to the *7SL* RNA. Experiments were done at least four times. (B) Total levels of *AATP11* and *PT-X* in control or stressed cells were visualized by Northern blot analysis using specific radioactive probes [19]. RNA samples were extracted from cells starved during 3 hours in PBS or incubated in the presence of 50 µM sodium arsenite for 1–4 hours.

As shown above, stress modulated both the localization of and the proteins associated with DRBD3. Since DRBD3 binds to a specific subset of mRNAs promoting their stabilization [19], [20], we next analyzed if DRBD3 remains associated with its target mRNAs upon stress. The levels of three DRBD3-bound mRNAs were measured after immunoprecipitation of DRBD3 ribonucleoprotein particles followed by quantitative RT-PCR. As shown in Figure 5A, mRNAs encoding AATP11 (amino acid transporter 11), PT-X (pteridine transporter on chromosome X) or NT10 (nucleobase transporter 10) remained bound to DRBD3 after starvation or arsenite treatment. Total cellular levels of *AATP11* and *PT-X*, as measured by Northern blot analysis, decreased 1.7±0.2–fold and 2.3±0.4–fold, respectively, upon incubation with arsenite for 3 hours (n = 3) but remained unchanged (1.2±0.1 and 1.1±0.1-fold, n = 3) in starvation conditions (Figure 5B).

## Discussion

Trypanosomes undergo profound morphological and biochemical changes to adapt to the different environmental conditions in the various niches they occupy in mammalian and insect hosts. Up to 30% of the transcriptome is remodeled during differentiation from mammalian bloodstream trypanosomes to procyclic insect forms [25], [26], [27], [28]. Since transcription initiation is not selectively regulated, it has been proposed that RNA-binding proteins (RBPs) act as ‘post-transcription factors’ that are at the end of signaling cascades linking environmental cues to mRNA and protein abundance [3], [7]. Indeed, several reports have highlighted the importance of signaling pathways in regulating differentiation and proliferation in trypanosomes [29]. However, it is still unknown how RBPs transduce environmental information into mRNA and protein abundance. DRBD3 is one of the few characterized RBPs in these parasites. It binds to a subset of mRNAs and promotes their stabilization [19], [20]. In this report we investigated whether DRBD3 ribonucleoprotein complexes respond to changes in the environment. In normal culture conditions, DRBD3 resides mainly in the cytoplasm. Upon arsenite-induced stress, however, DRBD3 concentrates in the nucleus. A similar behavior has been described for the RBPs UBP1 and UBP2 in *T. cruzi* [15]. This phenomenon seems to be specific to a subset of RBPs, as other RBPs do not translocate to the nucleus under the same conditions (Figure S2 and [15]). The behavior of DRBD3 is consistent with a function as a shuttle protein that transports target mRNAs from the nucleus to the cytosol. In fact, a fraction of DRBD3 is also detected in the nucleus of unstressed cells (Figure 1 and [19]). In another study, DRBD3 (named PTB1 in that report) showed a prominent nuclear localization under standard growth conditions [20]. As observed for several other RBPs in *T. cruzi* [18], starvation induces the accumulation of DRBD3 in stress granules (SGs), as judged by co-localization with the SG marker SCD6. In eukaryotes, SGs are thought to accumulate translationally-stalled mRNAs which are protected from degradation until environmental conditions improve [12]. This seems to be also the case in trypanosomes, since total levels of DRBD3-regulated mRNAs are unaltered upon starvation stress and remain bound to the complex.

How RBPs interact to other proteins to carry out their tasks is poorly understood in trypanosomes. Tandem-affinity purification (TAP) was performed to investigate how stress influences the protein composition of DRBD3 ribonucleoprotein complexes. DRBD3 was found to associate with several other proteins in unstressed cells forming a stable complex. The more abundant proteins in the complex are poly(A)-binding proteins PABP1/PABP2 and up to 20 different ribosomal proteins. Association with PABPs was found to be RNA-dependent, and was confirmed by detection of DRBD3 when TAP was performed using each PABP as bait. The functional consequences of this association are not known at present but, since DRBD3 is a stabilizing protein and PABPs protect mRNAs from degradation by deadenylases, it is possible that DRBD3 strengthens PABPs interaction with the mRNAs, as has been proposed for other RBPs in mammalian cells [30]. DRBD3 is the most abundant protein in the eluates. There are two possible explanations for this: there could be multiple complexes and/or the complexes partially or fully dissociate during the purification. Since the TAP purification procedure takes hours, the latter is likely to occur whether or not there are multiple complexes. This means that the purified material contains a mixture of different complexes, perhaps representing heterogeneity in the cell but also representing a mixture of complexes that have partially or fully dissociated during purification.

The presence of ribosomal proteins in the TAP-purified material suggests association to ribosomes. However, DRBD3 could not be detected in either polysomal fractions or TAP-purified ribosomes. Ribosomal proteins are typical contaminants present in many biochemical purifications, and indeed they have been detected in several pull-down experiments in *T. brucei*, albeit not in such abundance and number (reviewed in [31]). In addition, we performed TAP of the RBPs PABP2 and RRP44 and compared their patterns with those obtained with DRBD3 and ribosomes. As shown in Figure S3A, we did not observe any apparent contamination of ribosomal proteins in either PABP2 or RRP44 purifications. TAP carried out with a cell line expressing only the TAP tag did not yield any ribosomal protein either (data not shown). Moreover, a very similar pattern of ribosomal proteins associated with DRBD3 was consistently observed in up to eight independent TAP purifications (Figure S3B). The presence of ribosomal proteins in the purified TAP-DRBD3 could be explained if DRBD3 is not physically interacting with ribosomes, but located in close proximity to them, for example near the rough endoplasmic reticulum, forming cytoplasmic substructures that remain aggregated during the purification process. This is supported by the fact that no ribosomal proteins copurified with DRBD3 upon arsenite treatment, when most DRBD3 is located in the nucleus.

Under stress conditions, we found that target mRNAs remain bound to DRBD3. Total levels of two target transcripts, *AATP11* and *PT-X*, are decreased about 2-fold upon arsenite treatment. Since DRBD3 stabilizes target transcripts [19], these results suggest that not all *AATP11* and *PT-X* molecules are bound to DRBD3 in the cell, and that the pool associated with DRBD3 is more protected from degradation in arsenite conditions. Indeed, it is known that most mRNAs associate to multiple RNA-binding proteins in other eukaryotes ([32] and references therein). Moreover, we have found that *PT-X* also binds to a different RNA-binding protein in trypanosomes (data not shown).

DRBD3 is dissociated from most proteins upon arsenite-induced stress, PABPs being the only proteins detected in the complex. This is in apparent contradiction to localization data since DRBD3, but not PABPs, translocated to the nucleus after arsenite treatment. However, a significant amount of DRBD3 remained in the cytoplasm in these conditions, where it could be associated with PABPs. Starvation, on the other hand, promotes the dissociation of PABP2, but not PABP1, and the binding of a HSP70 chaperone. All these changes indicate that DRBD3 complexes are remodeled in response to environmental stimuli. Interestingly, two splicing factors, U1-70K and U1C, copurify with DRBD3. Since depletion of DRBD3 causes splicing defects in target mRNAs [20], our results suggest that DRBD3 binds to target transcripts already in the nucleus, transports them to the cytoplasm and regulates their fate through the association to various proteins involved in RNA splicing and turnover.

## Materials and Methods

### Trypanosome Culture

*Trypanosoma brucei* Lister 427 procyclic cells were grown at 27°C in SDM-79 medium [33] containing 10% fetal bovine serum. Sodium arsenite was used at a final concentration of 50 µM. For starvation experiments, cells were collected from logarithmic cultures, washed twice in phosphate-buffered saline (PBS), resuspended in the original volume of PBS and incubated at 27°C for 3 h with gentle shaking. Transgenic trypanosomes were obtained following standard procedures [34].

### Immunofluorescence Assays

Immunolocalization studies were carried out as previously described [17], [19], using polyclonal anti-*Tb*DRBD3 [19], anti-protein A (Sigma) or BB2 monoclonal antibodies [24].

### Expression of TAP-, PTP-, eYFP-, mChFP- and Ty-fusion Proteins

Details of constructs for the expression of tagged proteins are provided in Figure S1, and are based in vectors described in [35]. Tagged proteins were expressed from their endogenous loci except for RRP44-TAP, which was inducibly expressed from a ribosomal DNA spacer using 1 µg/ml tetracycline for 48 hours.

### Tandem Affinity Purification (TAP)

Cells (1–2×10^10^) were harvested in log phase, washed in serum-free SDM-79 medium and frozen at −80°C until use. Protein extracts were obtained by resuspending the cell pellet in 1 ml of lysis buffer per 10^9^ cells [10 mM Tris-HCl, pH 7.6, 2 mM DTT, 0.1% (w/v) Igepal CA-630, Complete protease inhibitor cocktail without EDTA (Roche)] and passing the suspension through a 27-gauge syringe thrice on ice. Lysates were centrifuged at 16,000×*g* for 10 min at 4°C. NaCl was added to the supernatant at a final concentration of 150 mM. TAP procedure was performed as described in [21], except that TEV digestion was carried out overnight at 4°C, and binding to calmodulin beads was allowed to proceed for 4 hours at 4°C. Protein complexes were eluted in 1 ml of 10 mM Tris-HCl, pH 7.4, 150 mM NaCl, 20 mM EGTA, precipitated for 30 min on ice with 20% trichloroacetic acid and 0.08% sodium deoxycholate in siliconized tubes, washed with 100% acetone, air-dried, loaded in 10% PAGE-SDS gels and stained with Sypro Ruby. Protein bands were excised, subjected to MALDI-TOF analysis and identified using MASCOT software (http://www.matrixscience.com).

For RNAses treatment, half of the lysate was incubated with 50 µg of RNase A and 1,000 units of RNase T1 for 30 min on ice before centrifugation. To confirm proper digestion, RNA was extracted from TEV eluate aliquots. cDNA was obtained from 1 µg of DNAse-treated RNA using 0.5 µg of random hexamers (Invitrogen) and Maxima reverse transcriptase (Fermentas), and PCR-amplified using specific primers pairs for the mRNAs encoding the amino acid transporter AATP11 (Tb927.4.4730), the pteridine transporter on chromosome 10, PT-X (Tb927.10.9080), and the nucleobase transporter *Tb*NT10 (Tb927.9.7470), as described [19].

To confirm the association of DRBD3 with different protein partners, small-scale purifications were carried out from 3×10^9^ cells expressing candidate proteins fused to PTP or TAP tags. In this case, TEV eluates were precipitated with trichloroacetic acid as above, loaded in 10% SDS-PAGE gels, transferred to nitrocellulose membranes and assayed for the presence of DRBD3 using anti-DRBD3 antiserum [19].

### Polysome Analysis

The association of DRBD3 with polysomes was tested using 10%–50% sucrose gradients as previously described [17]. Both puromycin and cycloheximide were used at 50 µg/ml. EDTA was used at 50 mM.

### mRNA Analysis

The association of mRNAs with DRBD3 under different conditions was assayed by immunoprecipitation of DRBD3 ribonucleoprotein complexes followed by quantitative RT-PCR. Cell lysates were obtained from 1×10^9^ cells as above. Vanadyl ribonucleoside complexes (Sigma) and RiboLock (Fermentas) were included in the lysis buffer at a concentration of 2 mM and 50 U/ml, respectively. One half of the lysate was incubated with DRBD3 antiserum bound to protein-G Dynabeads, and the other half was incubated with normal rabbit serum (Sigma) as a control. Beads were washed four times in 10 mM Tris-HCl, pH 7.4, 150 mM NaCl, 0.1% (w/v) Igepal CA-630 and incubated for 30 min at 50°C with 100 µg of proteinase K in 10 mM Tris-HCl, pH 8, 100 mM NaCl, 0.5% SDS and 1 mM EDTA. RNA was extracted with phenol:chloroform and treated with RQ1 DNase I (Promega). 300 ng of RNA were converted to cDNA using random hexamers as above. Quantitative RT-PCR reactions were performed using Fermentas SYBR Green master mix in a BioRad CFX96 thermal cycler with the following cycling conditions: 95°C, 10 min; 40 × [95°C, 15 s; 55°C, 30 s]. To confirm specificity, amplification was followed by melting temperature analysis, agarose gel electrophoresis and DNA sequencing. In addition, minus reverse transcriptase controls were included to rule out contamination of genomic DNA. Data were normalized relative to amplification of the signal recognition particle *7SL* RNA. The following primers were used:

*AATP11:* AE184 (CAAACAGCCCTTATTCAACACCTCACG), AE215 (GCAGCTCAGTGTCTCTATAAATCAATGC); *PT-X*: AE126 (GCTGAAGTGCAGGAGAGCGGTAG), AE186 (GTTTTTGCTCGCAACAACTTCTACG); *TbNT10*: AE647 (GAGAGCATGTTAACTCAGTCGAGG), AE648 (GCTGCAGCATGCCTAAACTCAGC); *7SL*: AE460 (AGCCGGAGCGCATTGCTCTG), AE461 (CAACACCGACACGCAACC).

## Supporting Information

Figure S1 Plasmids used in this work. Constructs (A) and strategy (B) used to generate a cell line expressing TAP-DRBD3 from the endogenous locus. (C) Assessment of DRBD3 expression in wild-type, DRBD3/− and TAP-DRBD3/− cell lines. One *DRBD3* allele was replaced by a blasticidin resistance marker using plasmid pGR139. The resulting cell line, DRBD3/−, expressed a reduced amount of endogenous DRBD3. DRBD3/− cells were transfected with plasmid pGR136 to generate TAP-DRBD3/− trypanosomes that no longer produced endogenous DRBD3 and expressed a TAP-tagged version of DRBD3 instead. Incubation of protein extracts from TAP-DRBD3/− cells with TEV protease caused cleavage of the IgG domains from the TAP sequence and the appearance of a protein corresponding to DRBD3 fused to the calmodulin-binding peptide (CBP) of the TAP tag, as expected. The cytosolic marker CSM was used as a loading control. (D) List of plasmids used for the expression of tagged proteins. All constructs target the endogenous loci, except for pHD1360, which is inserted into the rDNA spacer locus. p3378 was described in [17]; pHD360 in [36]; pHD918 in [23]; all other parental plasmids were described in [35].
(TIF)

Figure S2 DHH1, PABP1 and PABP2 do not translocate to the nucleus upon arsenite treatment. Cells expressing 4xTy-tagged versions of these proteins were incubated with 50 µM sodium arsenite for 4 hours and processed for immunofluorescence analysis using both anti-DRBD3 antiserum and BB2 monoclonal antibodies. Bound antibodies were detected using Alexa Fluor 488 goat anti-rabbit and Alexa fluor 594 goat anti-mouse IgGs. The results obtained after 3 hours exposure to arsenite were undistinguishable from those shown here (data not shown).
(TIF)

Figure S3 Tandem-affinity purification (TAP) of PABP2, DRBD3, the ribosome and RRP44. (A) Contamination of ribosomal proteins in TAP-purified PABP2 or RRP44 was assessed by mass spectrometry of all the bands visualized by Sypro staining. TAP of DRBD3 and the ribosome (using the ribosomal protein L18 as bait) are included for comparison. None of the bands analyzed in PABP2 and RRP44 purifications corresponded to any ribosomal protein. Asterisks indicate the protein used as bait in each case. The ∼ 70 kDa band in the PABP2 purification corresponds to PABP2 fused to the calmodulin-binding peptide of the TAP tag; the ∼ 65 kDa band is endogenous PABP2, and the ∼ 55 kDa probably represents a PABP2 degradation product. The presence of both tagged and endogenous PABP2 molecules in the purified complex indicates self-association of PABP2 within the cell. (B) Association of DRBD3 to ribosomal proteins was consistently observed in eight independent TAP purifications.
(TIF)
