# Supplementary figures and images for: Alterations in DRBD3 Ribonucleoprotein Complexes in Response to Stress in Trypanosoma brucei
Source: PLoS One. 2012 Nov 8;7(11):e48870. doi: 10.1371/journal.pone.0048870 (PMC3493610; doi:10.1371/journal.pone.0048870)

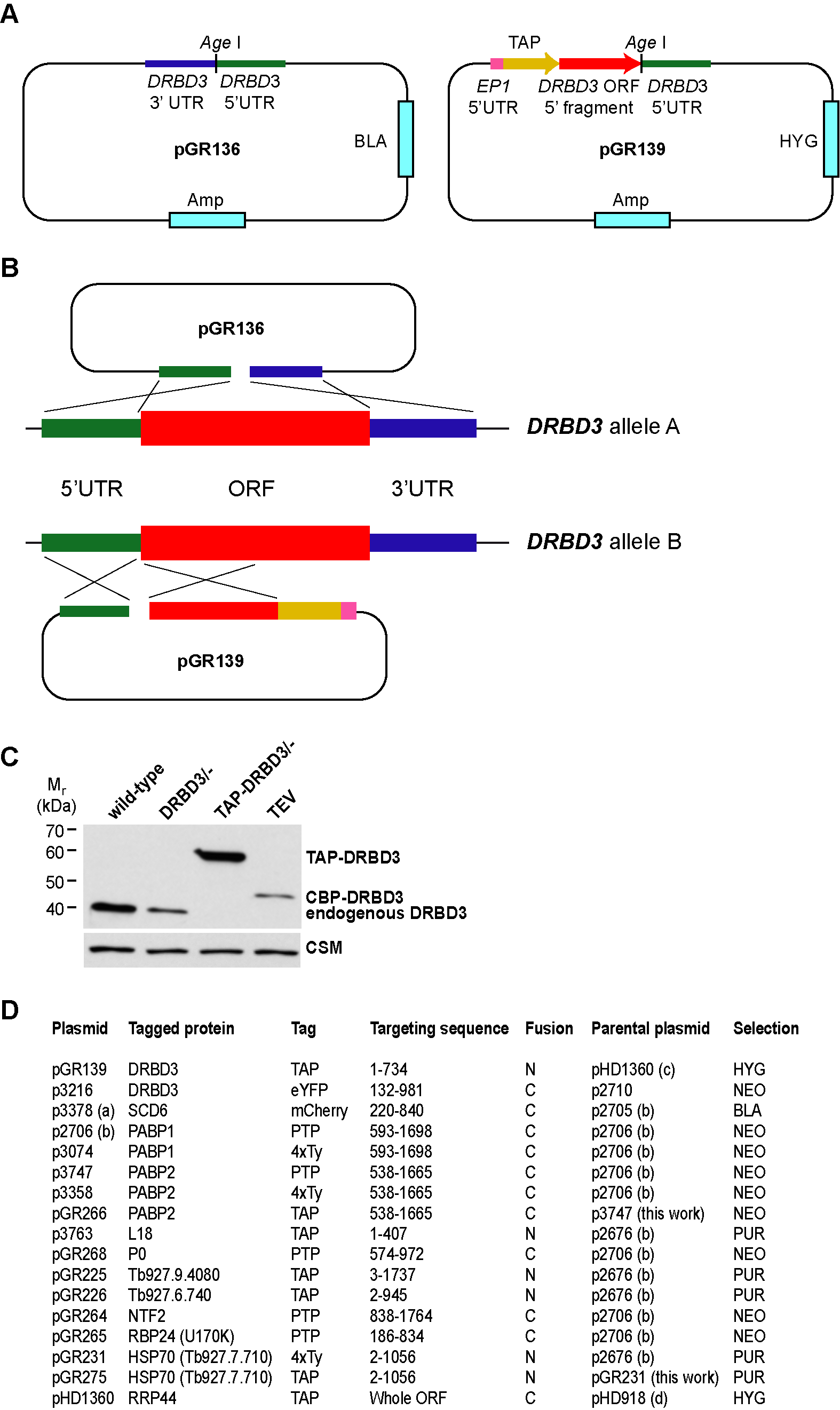

Supplement: Figure S1 — Plasmids used in this work. Constructs (A) and strategy (B) used to generate a cell line expressing TAP-DRBD3 from the endogenous locus. (C) Assessment of DRBD3 expression in wild-type, DRBD3/− and TAP-DRBD3/− cell lines. One DRBD3 allele was replaced by a blasticidin resistance marker using plasmid pGR139. The resulting cell line, DRBD3/−, expressed a reduced amount of endogenous DRBD3. DRBD3/− cells were transfected with plasmid pGR136 to generate TAP-DRBD3/− trypanosomes that no longer produced endogenous DRBD3 and expressed a TAP-tagged version of DRBD3 instead. Incubation of protein extracts from TAP-DRBD3/− cells with TEV protease caused cleavage of the IgG domains from the TAP sequence and the appearance of a protein corresponding to DRBD3 fused to the calmodulin-binding peptide (CBP) of the TAP tag, as expected. The cytosolic marker CSM was used as a loading control. (D) List of plasmids used for the expression of tagged proteins. All constructs target the endogenous loci, except for pHD1360, which is inserted into the rDNA spacer locus. p3378 was described in [17]; pHD360 in [36]; pHD918 in [23]; all other parental plasmids were described in [35]. (TIF) [file pone.0048870.s001.tif]

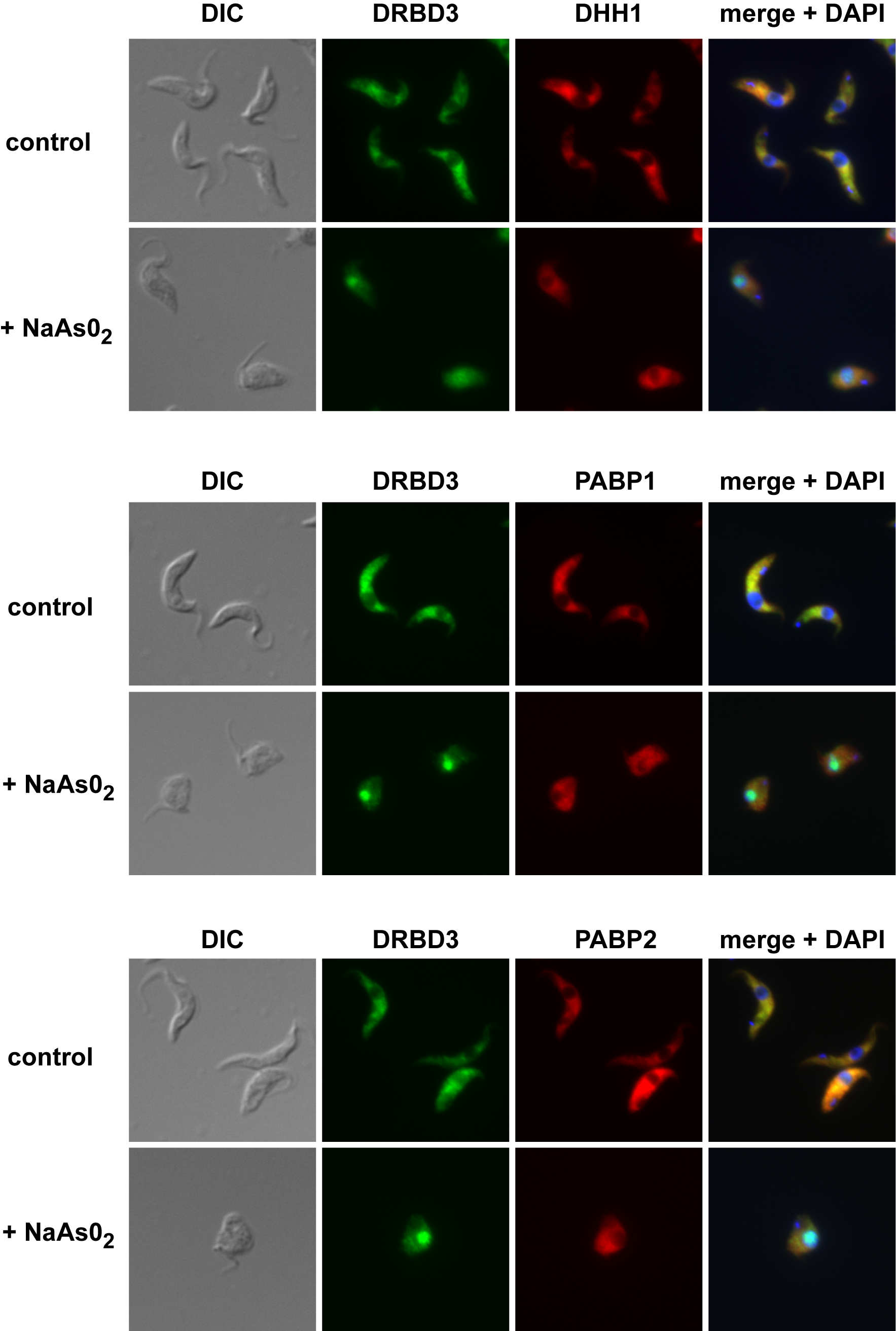

Supplement: Figure S2 — DHH1, PABP1 and PABP2 do not translocate to the nucleus upon arsenite treatment. Cells expressing 4xTy-tagged versions of these proteins were incubated with 50 µM sodium arsenite for 4 hours and processed for immunofluorescence analysis using both anti-DRBD3 antiserum and BB2 monoclonal antibodies. Bound antibodies were detected using Alexa Fluor 488 goat anti-rabbit and Alexa fluor 594 goat anti-mouse IgGs. The results obtained after 3 hours exposure to arsenite were undistinguishable from those shown here (data not shown). (TIF) [file pone.0048870.s002.tif]

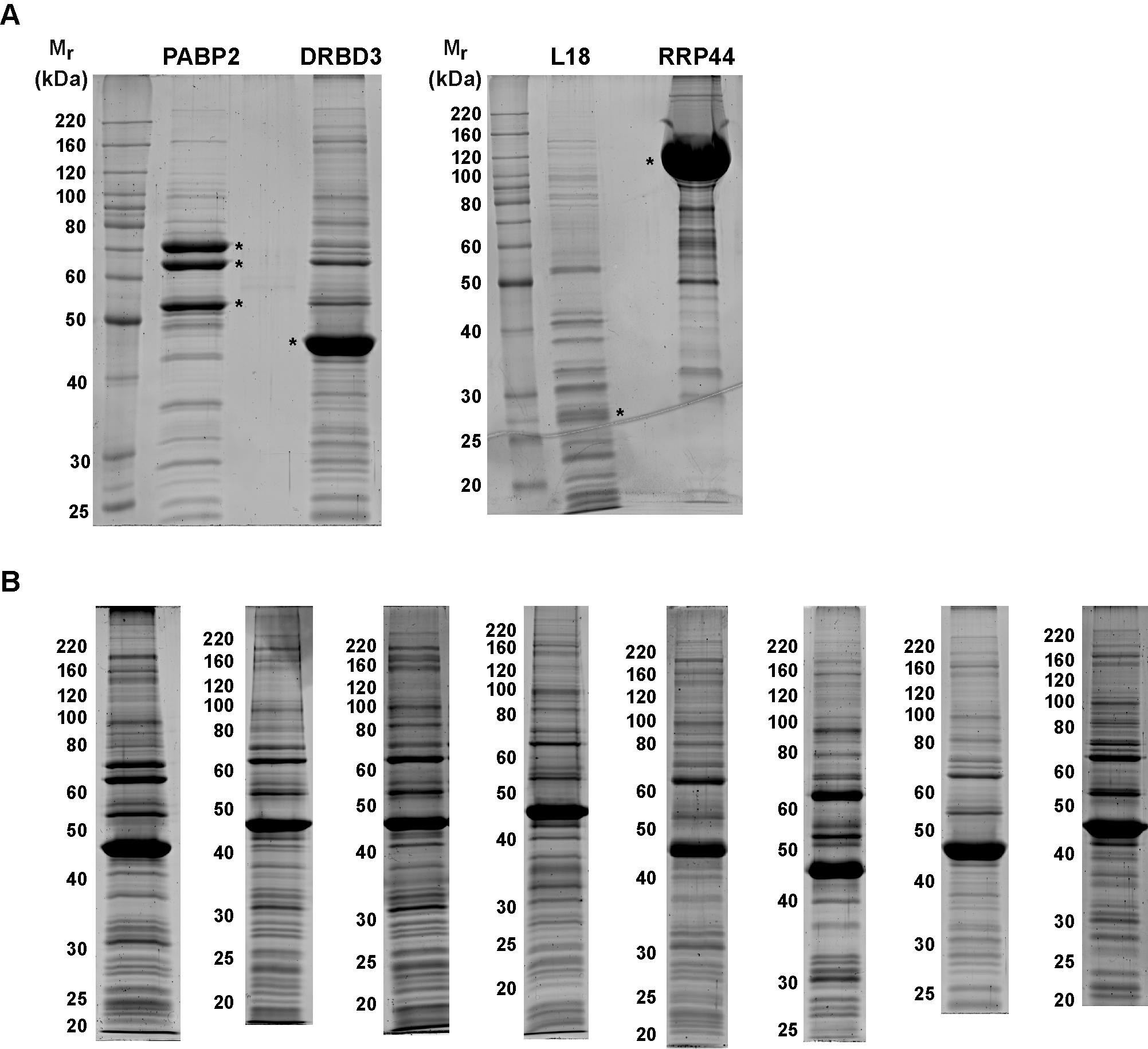

Supplement: Figure S3 — Tandem-affinity purification (TAP) of PABP2, DRBD3, the ribosome and RRP44. (A) Contamination of ribosomal proteins in TAP-purified PABP2 or RRP44 was assessed by mass spectrometry of all the bands visualized by Sypro staining. TAP of DRBD3 and the ribosome (using the ribosomal protein L18 as bait) are included for comparison. None of the bands analyzed in PABP2 and RRP44 purifications corresponded to any ribosomal protein. Asterisks indicate the protein used as bait in each case. The ∼ 70 kDa band in the PABP2 purification corresponds to PABP2 fused to the calmodulin-binding peptide of the TAP tag; the ∼ 65 kDa band is endogenous PABP2, and the ∼ 55 kDa probably represents a PABP2 degradation product. The presence of both tagged and endogenous PABP2 molecules in the purified complex indicates self-association of PABP2 within the cell. (B) Association of DRBD3 to ribosomal proteins was consistently observed in eight independent TAP purifications. (TIF) [file pone.0048870.s003.tif]
